# Supplementary material for: Growth inhibitory factor/metallothionein-3 is a sulfane sulfur-binding protein
Source: eLife. 2025 Nov 14;12:RP92120. doi: 10.7554/eLife.92120 (PMC12618007; doi:10.7554/eLife.92120)
Supplement: Figure 2—source data 1. [file elife-92120-fig2-data1.docx]

Figure 2-source data 1. Peak assignments for apo-GIF/MT-3 model structures.

| Wavelength | Raman Intensity | Assignment |
| --- | --- | --- |
| apo-GIF/MT-3_S4 | | |
| 403.8 | 87.7 | SS-SS (alpha), bent + NH bent |
| 410.6 | 164.0 | C-SS (alpha) bent + NH bent |
| 436.8 | 34.1 | SS-SS(beta), stretch |
| 462.7 | 78.5 | SSS-S (beta), bent |
| 464.2 | 29.7 | SSS-S(beta), stretch |
| 468.6 | 23.9 | SS-SS (alpha), stretch |
| 468.9 | 91.6 | SSS-S (alpha), stretch |
| 480.1 | 18.6 | SSS-S(beta), stretch |
| 482.9 | 45.3 | SS-SS(beta), bent |
| 485.7 | 36.2 | SSS-S (alpha), stretch |
| 489.3 | 12.0 | SSS-S (alpha), stretch |
| 489.6 | 32.3 | S-SH (beta) stretch+ methyl bent |
| 489.6 | 39.2 | S-SH (beta) stretch+ methyl bent |
| 490.8 | 33.2 | S-SH(alpha) stretch + methyl bent |
| 493.3 | 35.4 | S-SH(alpha) stretch + methyl bent |
| 493.6 | 52.5 | S-SH (beta) stretch+ methyl bent |
| 494.2 | 45.7 | S-SH (beta) stretch+ methyl bent |
| 497.8 | 13.9 | SSS-S (alpha), stretch |
| 501.6 | 39.4 | S-SH(alpha) stretch |
| 502.3 | 43.7 | S-SH (beta) stretch |
| 502.3 | 38.1 | S-SH(alpha) stretch |
| 506.1 | 43.6 | S-SH(alpha) stretch |
| 508.9 | 20.9 | SSS-S (alpha), stretch |
| apo-GIF/MT-3_S2 | | |
| 391.0 | 12.5 | SS stretch (alpha) + NH stretch |
| 413.8 | 40.6 | SS stretch (beta) |
| 472.3 | 11.0 | NH stretch (alpha) |
| 479.6 | 32.8 | SS stretch (beta) |
