# Supplementary material for: Growth inhibitory factor/metallothionein-3 is a sulfane sulfur-binding protein
Source: eLife. 2025 Nov 14;12:RP92120. doi: 10.7554/eLife.92120 (PMC12618007; doi:10.7554/eLife.92120)
Supplement: Figure 2—source data 2. [file elife-92120-fig2-data2.docx]

Figure 2-source data 2. Peak assignments for Zn_7_S_20_GIF/MT-3 and Zn_7_GIF/MT-3 model structures.

| Wavelength | Raman Intensity | Assignment |
| --- | --- | --- |
| Zn_7_S_20_GIF/MT-3 | | |
| 469.1 | 37.3 | SS stretch (beta) |
| 469.7 | 56.1 | SS stretch (beta) |
| 471.6 | 41.8 | SS stretch (beta) |
| 472.4 | 57.9 | SS stretch (beta) |
| 473.7 | 71.1 | SS stretch (beta) |
| 478.6 | 69.6 | SS stretch (beta) |
| 493.6 | 30.7 | SS stretch (beta) |
| 494.9 | 117.4 | SS stretch (beta) |
| 498.2 | 137.2 | SS stretch (beta) |
| 408.4 | 20.2 | CH3 torsion, peptide(alpha) |
| 412.5 | 18.4 | CH3 torsion, peptide(alpha) |
| 458.0 | 139.4 | SS stretch (alpha) |
| 462.4 | 39.7 | SS stretch (alpha) |
| 468.0 | 146.7 | SS stretch (alpha) |
| 468.1 | 33.6 | SS stretch (alpha) |
| 471.3 | 79.2 | SS stretch (alpha) |
| 471.7 | 66.4 | SS stretch (alpha) + NH stretch |
| 477.9 | 26.9 | SS stretch (alpha) + NH stretch |
| 486.1 | 34.2 | SS stretch (alpha) + CH3 torsion, peptide |
| 488.3 | 7.2 | CH3 torsion (alpha), peptide |
| 494.9 | 36.7 | SS stretch (alpha) |
| 497.2 | 48.5 | SS stretch (alpha) |
| 497.4 | 34.7 | SS stretch (alpha) |
| 499.1 | 32.8 | NH stretch (alpha) |
| 515.6 | 2.4 | CH3 torsion, peptide(alpha) |
| Zn_7_GIF/MT-3 | | |
| 408.2881 | 4.0012 | CH3 torsion, peptide(alpha) |
| 419.7967 | 4.1649 | CH3 torsion, peptide(alpha) |
| 427.6395 | 3.8385 | CH3 torsion, peptide(alpha) |
| 491.5289 | 0.7488 | CH3 torsion, peptide(alpha) |
| 492.0817 | 3.461 | CH3 torsion, peptide(alpha) |
| 495.6339 | 6.3352 | CH3 torsion, peptide(alpha) |
|  |  |  |
