# Supplementary material for: Growth inhibitory factor/metallothionein-3 is a sulfane sulfur-binding protein
Source: eLife. 2025 Nov 14;12:RP92120. doi: 10.7554/eLife.92120 (PMC12618007; doi:10.7554/eLife.92120)
Supplement: Figure 9—source data 1. [file elife-92120-fig9-data1.docx]

**Figure 9-source data 1. Thermostability score of sulfane sulfur-bound MT isoforms with or without Zn.** Values were calculated using the Protein Design module in MOE. ∆Stability indicates the energy difference between MT with and without sulfane sulfur.

|  | Sulfane sulfur | Stability (kcal/mol) | ∆Stability (kcal/mol) |
| --- | --- | --- | --- |
| Zn_7_MT1 |  | -261 |  |
| Zn_7_MT2 |  | -259 |  |
| Zn_7_GIF/MT3 |  | -302 |  |
| Zn_7_MT1 | 20 | -368 | -107 |
| Zn_7_MT2 | 20 | -365 | -106 |
| Zn_7_GIF/MT3 | 20 | -409 | -107 |
| apo-MT1 |  | -190 |  |
| apo-MT2 |  | -191 |  |
| apo-GIF/MT3 |  | -221 |  |
| apo-MT1 | 20 | -148 | 42 |
| apo-MT2 | 20 | -150 | 41 |
| apo-GIF/MT3 | 20 | -179 | 42 |
