# Supplementary material for: Growth inhibitory factor/metallothionein-3 is a sulfane sulfur-binding protein
Source: eLife. 2025 Nov 14;12:RP92120. doi: 10.7554/eLife.92120 (PMC12618007; doi:10.7554/eLife.92120)
Supplement: Figure 10—source data 1. [file elife-92120-fig10-data1.docx]

Figure 10-source data 1. Fragment sequences of a mouse brain sulfane sulfur-binding protein, determined using nano-UPLC-MS.

| Position | Observed MS (Da) | Calculated MS (Da) | Sequence |
| --- | --- | --- | --- |
| 2-19 | 2028.75 | 2028.74 | DPETCPCPTGGSCTCSDK  + 4 Carbamidomethyl C |
| 33-44 | 1475.48 | 1574.48 | SCCSCCPAGCEK  + 5 Carbamidomethyl C |
| 48-63 | 1780.78 | 1780.78 | DCVCKGEEGAKAEAEK  + 2 Carbamidomethyl C |
